# Anterior segment optical coherence tomography imaging in peripheral ulcerative keratitis, a corneal structural description

**DOI:** 10.1186/s12886-020-01466-1

**Published:** 2020-05-25

**Authors:** Clemence Bonnet, Louis Debillon, Saba Al-Hashimi, Florence Hoogewoud, Dominique Monnet, Jean-Louis Bourges, Antoine Brézin

**Affiliations:** 1 Ophthalmology Department, Cochin Hospital, Paris University, Paris, France; 2 Stein Eye Institute, David Geffen School of Medicine, University of California in Los Angeles, 100 Stein Plaza, Los Angeles, CA 90095 USA

**Keywords:** Peripheral ulcerative keratitis, Anterior segment optical coherence tomography, Cornea, Systemic vasculitis

## Abstract

**Background:**

Anterior segment optical coherence tomography (AS OCT) is a helpful tool used to diagnose and manage many corneal conditions, but its use has not been reported in case of peripheral ulcerative keratitis (PUK). The aim of this study is to describe AS OCT findings in cases of PUK.

**Methods:**

Retrospective observational case series of six eyes presenting with a PUK and proven systemic vasculitis. Clinical course, slit lamp photographs, and AS OCT findings were the main outcomes.

**Results:**

The AS OCT findings were found to correlate with the ocular disease’s level of activity. In the acute stage, an absence of corneal epithelium, a scrambled appearance of the anterior stroma and a heterogeneous stromal reflectivity were observed. During the reduction of disease level activity, an irregular hyporeflective epithelium, a smoother anterior stroma, and a homogenous hyperreflective stroma were seen. At the healed stage, a filling of the corneal defect by a hyporeflective thick epithelium, the persistence of the hyperreflective underlying stroma, and a demarcation line were observed. The mean total corneal thickness at last follow-up was significantly thicker (509 ± 147 μm) compared with the mean corneal thickness at onset (408 ± 131 μm; *P* = 0.03).

**Conclusions:**

AS OCT provides an assessment of structural changes occurring in PUK, useful for its diagnosis and monitoring.

## Background

Peripheral ulcerative keratitis (PUK) is a rare form of corneal inflammation leading to corneal ulceration and stromal destruction. Its incidence is reported as three cases per million per year [1]. The most critical ophthalmic complication is corneal perforation which can occur quickly once the inflammation begins [2, 3]. In addition to the high risk of vision loss, a PUK is a harbinger of active systemic vasculitis, with high risk of morbidity and mortality [4]. Systemic glucocorticoids have been the basis of therapy for noninfectious PUK and additional immunosuppressive agents are usually used to prevent perforation and allow for a transition to a steroid sparing agent. The indication and dosing of systemic glucocorticoid steroid are empirical, as there is no consensus regarding the type of the drugs, their dosage and the duration of treatment [5]. Corneal structural changes can be at the forefront of the systemic disease [6]. The clinical diagnosis and the follow-up of PUK remain challenging, supported by only a few publications [7].

Anterior segment optical coherence tomography (AS OCT) is a relatively new imaging modality providing a transformative shift in the imaging field towards a better evaluation, diagnosis and management of many anterior segment diseases [8, 9]. The technology has evolved over the years, and a detailed evaluation of anterior segment structures, with finer details than slit lamp biomicroscopy, is achievable in a fast, non-contact and safe procedure [10]. Current reported uses of AS OCT are corneal thickness evaluation, depth of corneal flaps, depth of corneal deposits and lesions including dystrophies, details of corneal inflammation and Descemet’s membrane, dry eye evaluation and diagnosis of surface neoplasia in early stages [10]. The purpose of this study is to describe the AS OCT cornea features during active stage of PUK and to evaluate its contribution for its diagnosis and follow-up. Images acquired by AS OCT were compared to the clinical photographs to define optical coherence tomography diagnosis criteria of PUK.

## Methods

This retrospective descriptive monocentric case series included patients who presented with a PUK between December 2011 and October 2018 to the tertiary Ophthalmology Department at Cochin Hospital in Paris, France. At baseline, the diagnosis was made at slit lamp examination by two cornea specialists (C.B, F.H.) according to the commonly applied definition of PUK, i.e. the presence of a crescent-shaped destructive inflammatory process of the corneal stroma within the peri-limbic area, associated with an epithelial defect, the presence of stromal inflammatory cells and stromal keratolysis [6]. Associated scleritis and episcleritis or any anterior chamber inflammation were also noted. The research protocol was approved by the institutional human experimentation committee (IRB# 00008855). Written informed consent for the data collection and analysis was obtained from each patient. The study adhered to the tenets of the Declaration of Helsinki.

### Data collection

Data collected at baseline, 1 week, 1 month and 3 months were demographics, best corrected distance visual acuity (CDVA) with Snellen chart, slit lamp examination with fluorescein staining, anterior segment photography, and AS OCT. The clinical evaluation of corneal healing as a main result of treatment efficacy was based on non-progression of the corneal thinning, filling of the ulcer crater with negative fluorescein staining and a decrease of pain and ocular discharge. Recurrence or unresponsiveness to treatment were defined as persistent pain and ocular discharge, progression of the corneal thinning, and persistent fluorescein staining. Using AS OCT, the disease activity was monitored by the evaluation of the corneal thickness at the thinnest point and structural changes of the corneal ulcer. Specific anatomic features observed on AS OCT at each stage of the disease were outlined. In parallel, detailed medical history including previous systemic disease, and treatment were recorded. Physical examination along with complete serologic evaluation were performed at baseline to detect an underlying infectious or auto-immune condition.

### Images acquisition and measurement

Slit lamp photographs of the cornea were performed using the Canon 20D camera with a Haag-Streit flash unit for anterior segment photography (acquisition software: Eye Cap v7) and AS OCT was performed with the anterior segment module of Spectralis OCT (Heidelberg Engineering, Heidelberg, Germany). The wavelength was 870 nm, the A-line rate was 24 kHz with an axial resolution of 7 μm. At each time-point, 5 to 10 horizontal scans of 12-mm were acquired at a rate of 40 frames per scan [9]. The scanning probe was tilted in order to bring the scanning beam as perpendicular as possible to the lesion. On AS OCT scans, measurements of epithelial and stromal corneal thickness were performed using *ImageJ* software 1.52 version (National Institute of Health, USA). The measurements were performed at the thinnest point at onset. The caliper was placed perpendicular to the corneal endothelium. At last follow-up, the measurements were made at the same location, following the same protocol. Visual verification was performed on the infrared image provided by the AS OCT device by the cornea specialist (CB) who performed the measurement, to ensure that the same location was used at last follow-up.

### Statistical analysis

Visual acuities were converted to LogMAR for statistical analysis. Wilcoxon paired test was used for continuous data comparison. XLSTAT Addinsoft, version 2018.55292 (Addinsoft, Paris, France) statistical and data analysis was used to perform the analysis.

## Results

### Demographics and treatment

A total of six eyes of six patients including four women (66.7%) and 2 men (33.3%) with a mean age at diagnosis of 50.3 ± 14.7 years (range 33 to 74) and a mean follow-up of 4.4 ± 3.8 months were analyzed. The Table 1 summarizes the demographics, clinical examination at baseline, diagnosis and treatment for each patient. At onset, three patients had a previous diagnosis of systemic vasculitis: two had rheumatoid arthritis and one relapsing polychondritis. Laboratory work-up performed in the three other patients resulted in a diagnosis of a cryoglobulinemia type 2B, a granulomatosis with polyangiitis and a biologic connective-tissue disease. All patients were treated with intravenous pulses of methylprednisolone during days 1 to 5, transitioned to oral prednisone (1 mg/kg/day) and tapered up to 6 months. In cases of known systemic vasculitis, steroid sparing therapy was adjusted or switched as needed. In cases of recurrence under the first line of immunosuppression, cyclophosphamide (600 mg/m^2^) pulses were added until healing of PUK. Topical therapy consisted of steroids drops (dexamethasone, 1 mg/ml) three times a day tapered for 3 months, antibiotic drops (azithromycin dihydrate 15 mg/g) two times a day for 3 months and artificial tears (sodium hyaluronate) as needed. All patients were treated medically, no significant change in CDVA (*P* = 0.18) was noted and no cases of perforation or death were observed.

**Table 1:** Demographics, clinical examination at baseline, diagnosis and treatment

ID	Sex	Eye	Age at diagnosis	Associated symptoms	Initial CDVA (LogMAR)	Final CDVA (LogMAR)	Previous known systemic vasculitis	Previous systemic treatment	New diagnosis of systemic vasculitis	Intraveinous corticosteroids pulses (1 g), n	Steroid-sparing first line therapy	Recurrence	Second line therapy
1	M	OD	45	–	0.7	1	Rheumatoid arthritis	Rituximab	No	1	Tapered oral corticosteroids + Rituximab	No	No
2	F	OS	38	–	1	1	Relapsing polychondritis	No	No	3	Tapered oral corticosteroids + methotrexate	Yes	Cyclophosphamide
3	F	OS	74	–	0.4	0.4	No	No	Cryoglobulinemia type 2B	3	Tapered oral corticosteroids + Infliximab	No	No
4	F	OS	56	Scleritis	–	–	Rheumatoid arthritis	Methotrexate, Etanercept	No	3	Tapered oral corticosteroids + Rituximab	No	No
5	F	OD	54	Scleritis, Episcleritis	1	1	No	No	Granulomatosis with polyangiitis	5	Tapered oral corticosteroids	Yes	Cyclophosphamide
6	M	OS	33	–	0.4	0.9	No	No	Connective-tissue disease	4	Tapered oral corticosteroids + methotrexate	Yes	Cyclophosphamide
Mean ± SD					0.7 ± 0.3	0.9 ± 0.3							
*P* value *						0.18							

*CDVA* Corrected distance visual acuity, *ID* Patient study number, *n* number, *SD* standard deviation. *: Wilcoxon paired test

### Slit lamp examination

**Fig. 1:**
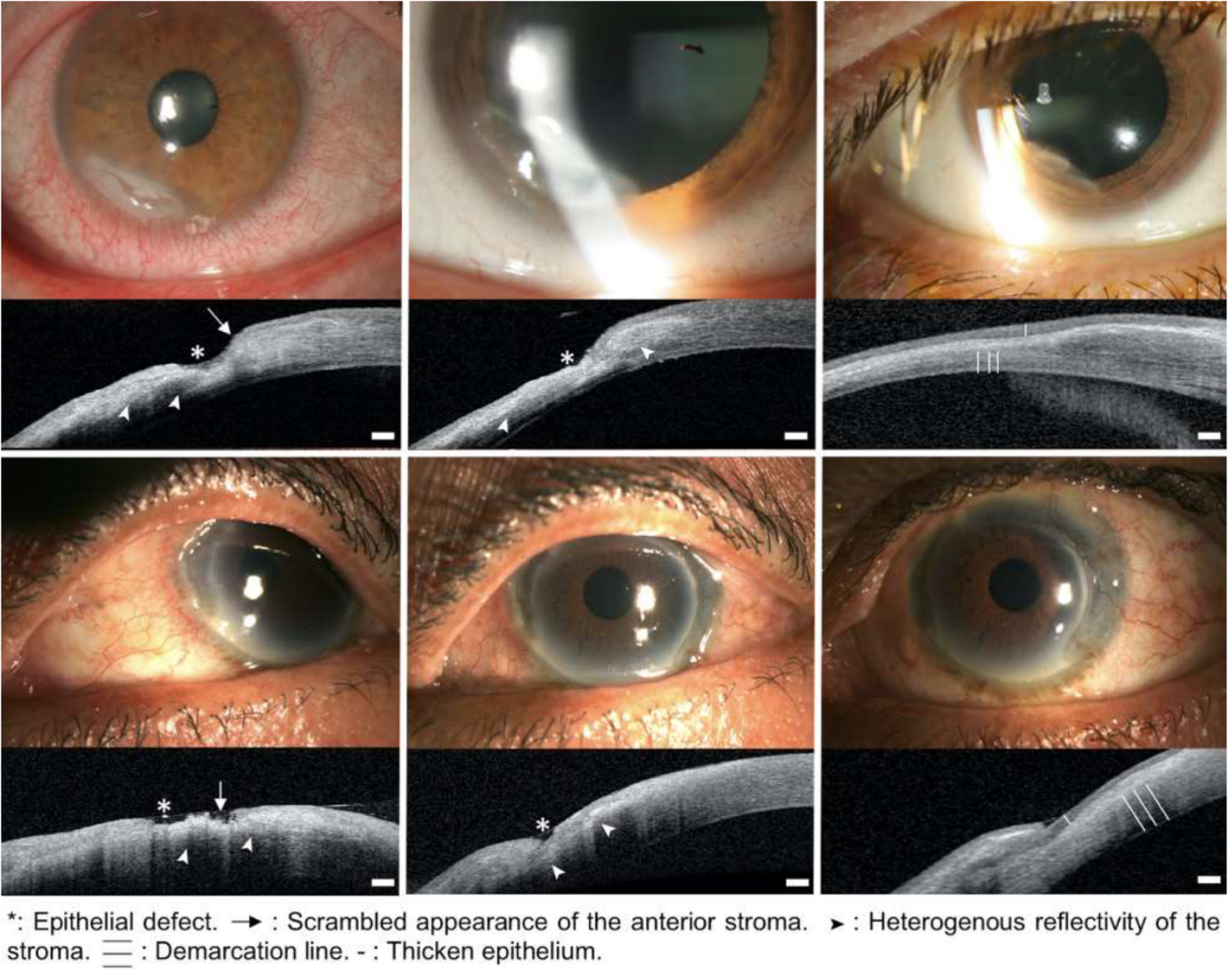
Slit lamp examination and AS OCT in 2 cases of good outcome under treatment. Left: active stage (baseline). Center: healing stage (day 7 to 21). Right: healed stage (1 to 3 months after the baseline examination). Top: first case, middle: second case. Scale bar 200 μm

Figure 1 shows the clinical aspects of PUK on slit lamp examination, and AS OCT scans, in cases of ulcer healing under the first line of systemic treatment. Corneal thickness stabilization, corneal ulceration healing and development of scar tissue along with decrease in pain, and resolution of scleritis and episcleritis were seen.

### AS OCT

#### Thinning

In all cases, thinning of the cornea was visible with achievable objective measurements of the remaining posterior stroma (Fig. 1). The mean total corneal thickness at last follow-up was significantly thicker (509 ± 147 μm) compared with the mean corneal thickness at onset (408 ± 131 μm; *P* = 0.03) (Table 2). There was no statistical difference in the mean stromal corneal thicknesses (433 ± 130 μm at last follow-up vs. 399 ± 126 μm at onset, *P* = 0.62). The epithelial thickness was often not measurable at onset given the epithelium was absent due to an epithelial defect when measured at the thinnest point.

**Table 2:** Corneal thickness at onset and last follow-up

ID	Epithelium thickness (μm)		Stromal thickness (μm)		Total corneal thickness (μm)	
	Onset	Last F/u	Onset	Last F/u	Onset	Last F/u
1	–	195	379	389	379	584
2	53	80	440	504	493	584
3	–	109	433	490	433	599
4	–	44	365	380	365	424
5	–	114	581	606	581	620
6	–	15	195	230	195	245
Mean ± SD	–	93 ± 63	399 ± 126	433 ± 130	408 ± 131	509 ± 147
*P* value*		–		0.62		0.03

*SD* standard deviation, *μm* microns. *Wilcoxon paired test, mean onset versus mean last follow-up thickness

#### Images description

**Fig. 2:**
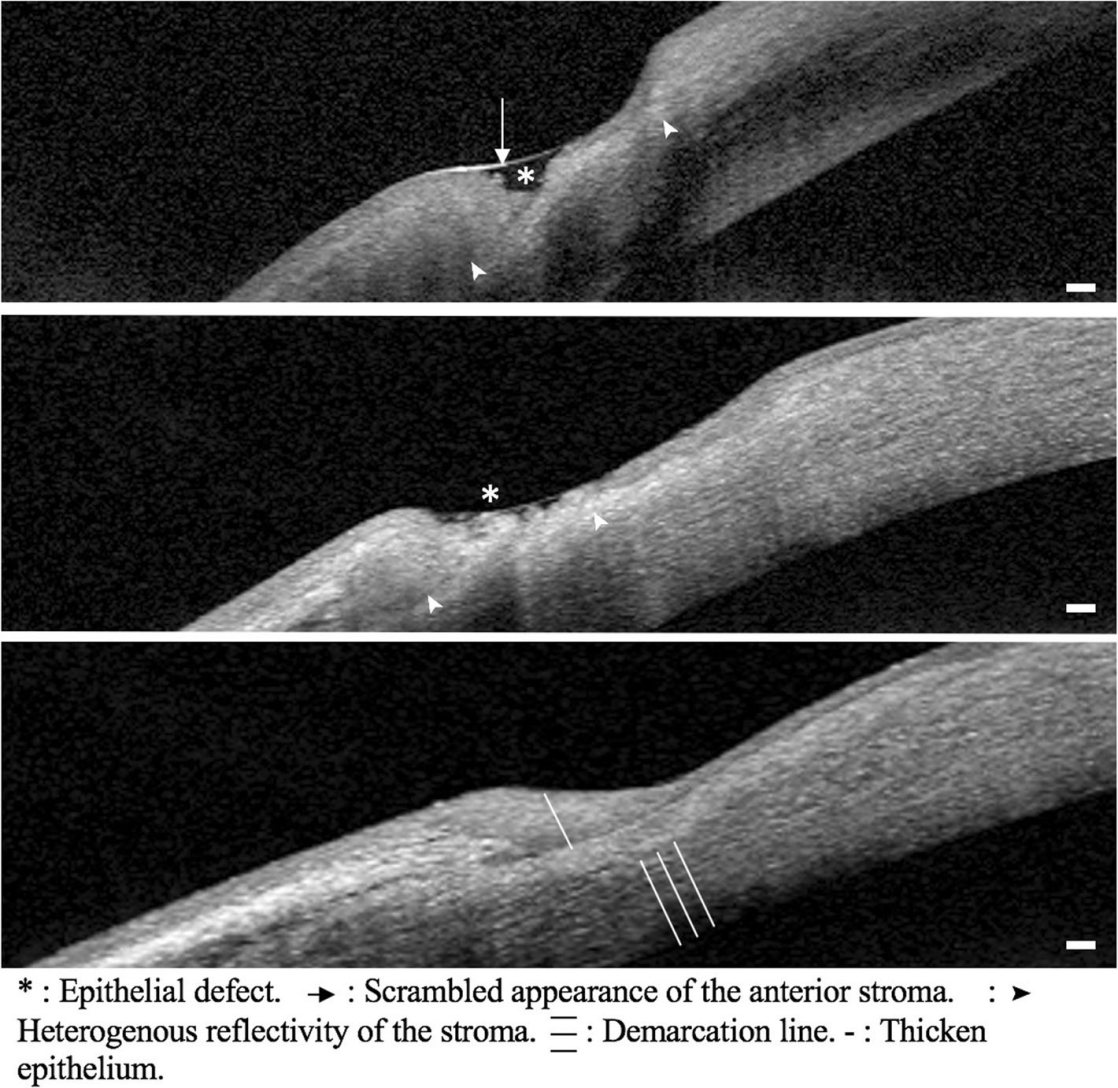
High magnification of AS OCT findings in peripheral ulcerative keratitis (case #3). Top: Active stage. Center: Healing stage. Bottom: Healed stage. Scale bar 200 μm

At the initial evaluation, AS OCT findings of active disease were: 1) The epithelial layer was not visible, 2) The surface of the anterior stroma was irregular with a scrambled appearance, 3) The reflectivity of the underlying stroma was variable - iso, hyper or hyporeflective, disorganized and heterogenous. After an initial intense systemic therapy, between day 7 and 21, AS OCT findings showed healing patterns: 1) The epithelial layer was irregular and hyporeflective, 2) The anterior stroma was smoother with a resolution of the scrambled appearance, 3) The reflectivity of the underlying stroma was more homogenous and hyperreflective. After 1 month, the AS OCT findings showed healed patterns: 1) The epithelial layer was hyporeflective and thicker, filling the area of corneal thinning, 2) The underlying stroma was hyperreflective and regular, 3) A demarcation line between the healed and unaffected stroma was visible (Fig. 2).

**Fig. 3:**
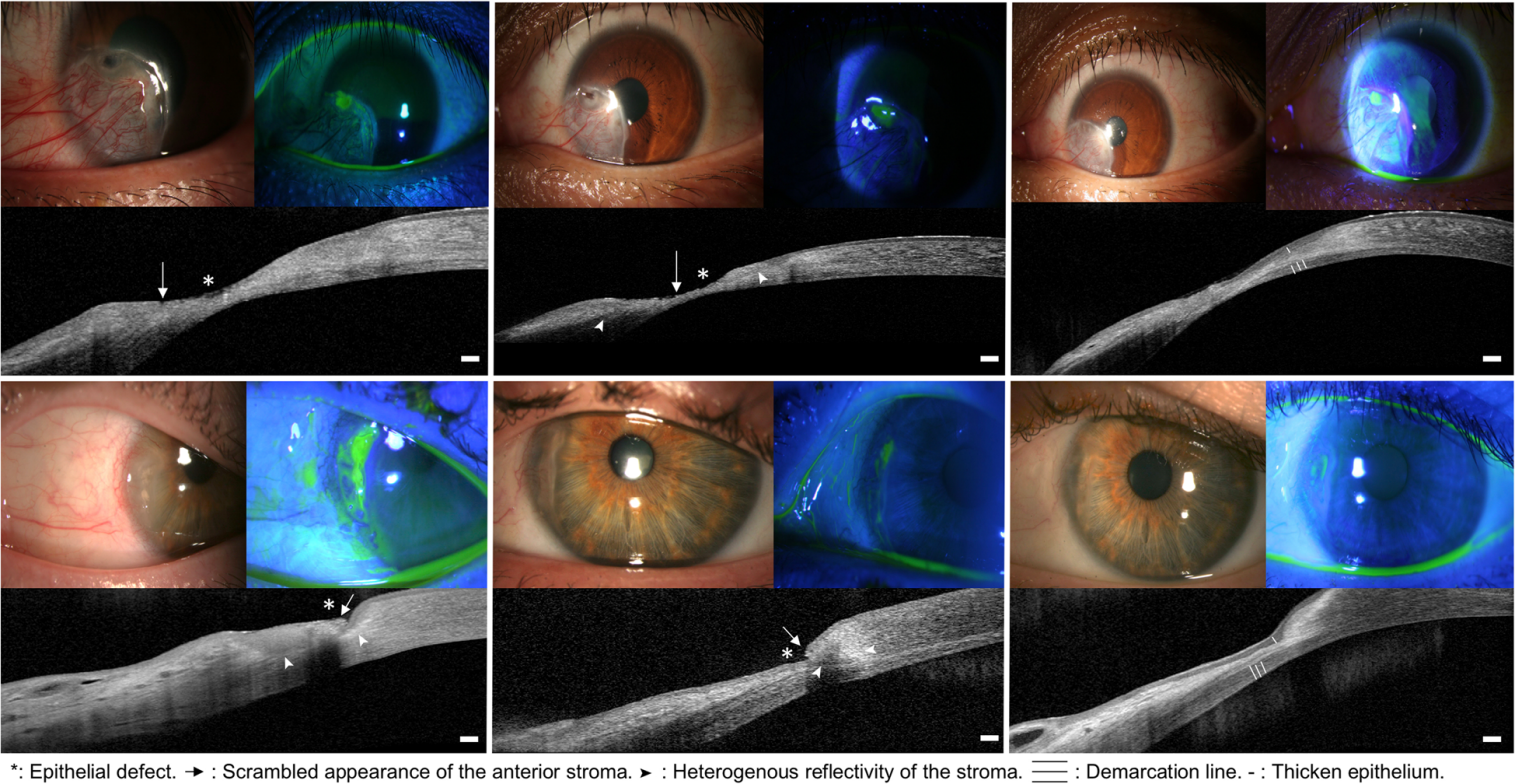
Slit lamp examination and AS OCT in 2 cases of insufficient outcome under treatment and recurrence. Left: active stage (baseline). Center: improvement (top, day 15) and recurrence (bottom, day 7). Right: healed stage (top and bottom, 1 to 3 months after the recurrence). Top: first case, bottom: second case. Scale bar 200 μm

In three cases, the same AS OCT findings than the one seen during the initial active stage were retrieved during the follow-up, and used to diagnose a recurrence of the PUK: an epithelial defect, an irregular scrambled anterior stromal surface, a heterogenous reflectivity of the remaining stroma and a progressive corneal thinning. After treatment intensification, the same AS OCT findings than the one seen at healed stage were retrieved: a thick intact epithelium, hyperreflectivity of the remaining stroma and a demarcation line (Fig. 3), confirming the healing process.

## Discussion

The gold standard to diagnose PUK is currently with slit lamp biomicroscopy [1]. Our study demonstrates the benefits of AS-OCT in the diagnosis and the monitoring of PUK, assessing morphologic changes that may be too subtle to be seen clinically. The clinical evaluation at an early stage of PUK can be very challenging; however, the high imaging resolution associated with the use of automated epithelial and corneal thickness software provided by AS OCT may be useful and more sensitive than the clinical examination to detect subclinical changes. In cases of recurrence, clinical examination is challenging due to irregular fluorescein pooling in the area of a previous activity, mimicking a corneal ulcer or early stromal thinning, without clear objective clinical signs. The monitoring of the level of disease activity is meaningful, as PUK can be associated with life threatening situations in the context of systemic diseases. The follow-up monitoring of these morphological and thickness changes on AS OCT could also be more sensitive than the clinical examination, and may be made easier using eye-tracking software [9, 10]. This study was not intended to test the sensitivity and the specificity of AS OCT compared to the gold standard clinical examination, which remains to be established. Nevertheless, detecting discrete anatomical signs of increased disease activity before they manifested clinically led to an intensification of immunosuppressive therapy and prevent corneal perforation or death. In a field with a low level of evidence-based guidelines for the management of PUK, making therapeutic decisions based on AS OCT findings was helpful [4, 11, 12]. We therefore recommend the use of AS OCT as an adjunctive objective tool in the diagnosis and management of PUK.

Other peripheral corneal lesions, infectious or inflammatory, can mimic a PUK, and AS OCT has been shown to be helpful in allowing an accurate diagnosis. For example, its usefulness in the setting of infectious keratitis has been described, with specific AS OCT features such as retrocorneal plaques and stromal necrosis [13, 14]. This may help to make a diagnosis when the slit lamp examination itself remains limited because of corneal opacification [13]. Adding our specific findings, the evaluation of complex peripheral inflammatory disease process which are clinically difficult to differentiate, can be improved by AS OCT.

In our series the diagnosis of PUK was certain as laboratory work-up found a systemic vasculitis in all cases, whereas classically 50% of PUK cases have an associated collagen vascular disease [6]. Our findings are the first step towards a better classification using AS OCT of the peripheral corneal inflammatory lesions’ spectrum, making progress towards standardization of their diagnosis and management, a difficult goal in rare diseases.

AS OCT has become an important tool in the evaluation and management of many corneal and anterior segment diseases, allowing a detailed evaluation in a non-contact and safe way [9]. In cases of ocular surface lesions, close correlation between AS OCT and histopathology findings have confirmed that AS OCT could serve as an adjunctive diagnosis modality [14]. In cases of PUK, histological diagnosis is not easily accessible [15]. Confocal microscopy could also be helpful, with its resolution of 1 μm /pixel, as it could provide images comparable to histochemical methods, thus enabling the study of epithelial cells and stromal keratocytes [16]. In vivo examination could also be helpful in understanding the clinical relevance of the hyperreflective stromal demarcation line that we observed at healed stage. Compared with other corneal diseases, it could correspond to the transition zone between post-inflamed anterior corneal stroma and the unaffected posterior corneal stroma, and may result from the difference in refractive indices or reflection properties of affected versus unaffected corneal stroma [17].

Using these imaging techniques, a better understanding of the pathophysiology of PUK can be achieved. The mechanisms of keratolysis are complex and currently poorly understood. The peripheral cornea and limbus reside close to conjunctival blood vessels and lymphatic channels, and with more Langherans cells and C1 components than the central cornea, are an ideal area for immune complex deposition as signs of active collagen vascular disease [15]. These depositions could result in activation of metalloproteinases (MMP -type one, two and nine-) and collagenases by inflammatory cells and adjacent conjunctival tissue, and have been found in patients with PUK [18, 19]. Another proposed mechanism for development of PUK centers around the alterations in the conjunctival vascular structure. Varying degrees of vaso-occlusion of the episcleral and conjunctival vasculature have been demonstrated in patients with PUK [20]. This vasculopathy may lead to resorption of stromal tissue, resulting in peripheral corneal ulceration and necrosis [11]. The scrambled appearance at the anterior stroma seen on our AS OCT during the active stage could be either residual fragments of epithelial cells or stromal anterior keratocytes. Depending on the level of disease activity, this appearance could also be either digested epithelial cells or keratocytes by MMP, or ischemic necrotic cells by vaso-occlusion, or a combination of both mechanisms. Likely, the pathophysiology of PUK is multifactorial, and further studies with larger samples and prospective AS OCT image acquisition is needed to strengthen our understanding of the disease process and to further define the role of AS OCT in the diagnosis and management of PUK.

Conclusions that can be drawn are limited due to the retrospective data and small sample size. Thickness measurements were made manually and the reproducibility of the measurements between examinations were visually assessed. In the future, the use of AS OCT devices implemented with eye-tracking and automated epithelial and corneal thickness mapping softwares could help define its place and identify severity criteria and risk factors of recurrences. AS OCT allowed visualization of morphologic changes, but the sensitivity and specificity of AS OCT in the diagnosis and management of PUK remain to be established.

## Conclusion

We have shown AS OCT signs of PUK. The use of AS OCT was helpful in monitoring the disease activity through corneal thickness evolution and recognizing specific patterns. It allowed for an early diagnosis of recurrences with subsequent successful modification of treatment. This highlight the utility of AS OCT as an adjunctive modality that can provide non-invasive imaging guidelines for the diagnosis and management of PUK.

## Data Availability

The datasets used and analyzed during the current study are available from the corresponding author on reasonable request.

## Abbreviations

AS OCT: Anterior segment optical coherence tomography
CDVA: Corrected distance visual acuity
LogMAR: Logarithm of minimum angle of resolution
MMP: Metalloproteinases
PUK: Peripheral ulcerative keratitis
